# Supplementary figures and images for: Genome-Wide Identification of GYF-Domain Encoding Genes in Three Brassica Species and Their Expression Responding to Sclerotinia sclerotiorum in Brassica napus
Source: Genes (Basel). 2023 Jan 15;14(1):224. doi: 10.3390/genes14010224 (PMC9858701; doi:10.3390/genes14010224)

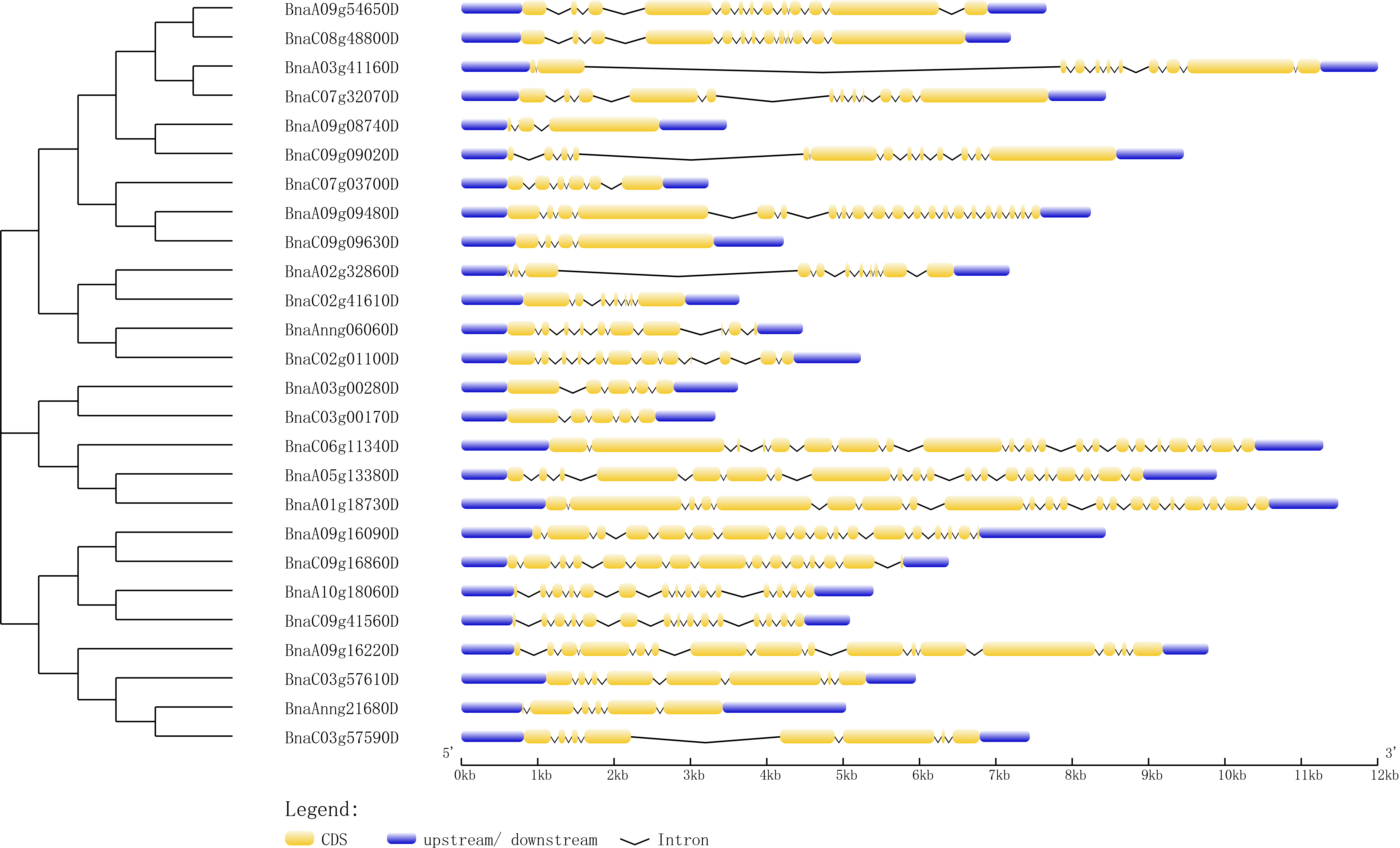

Supplement: Supplementary file 1 [file genes-14-00224-s001.zip › Supplementary figures/Figure S1.jpg]

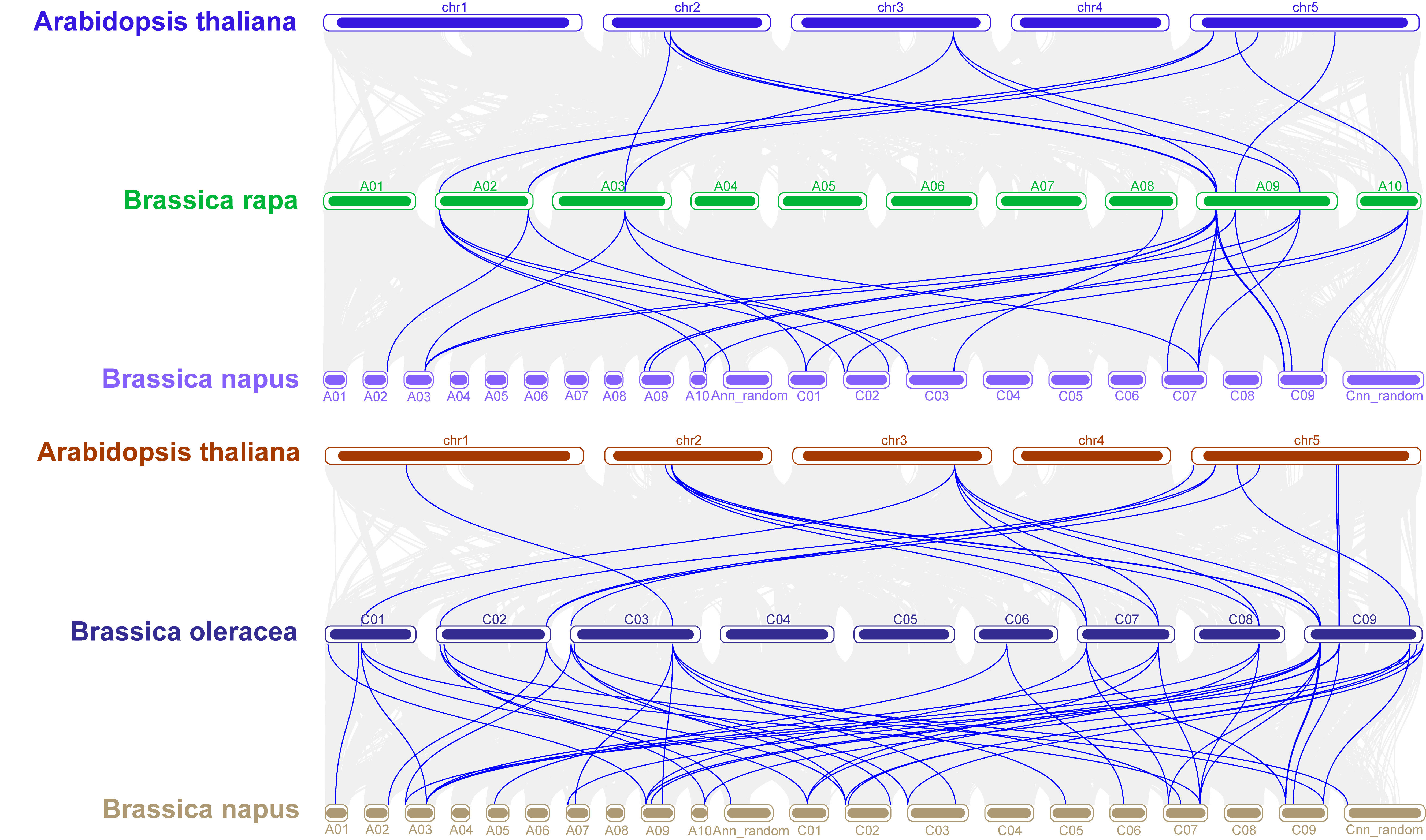

Supplement: Supplementary file 1 [file genes-14-00224-s001.zip › Supplementary figures/Figure S2.tif]

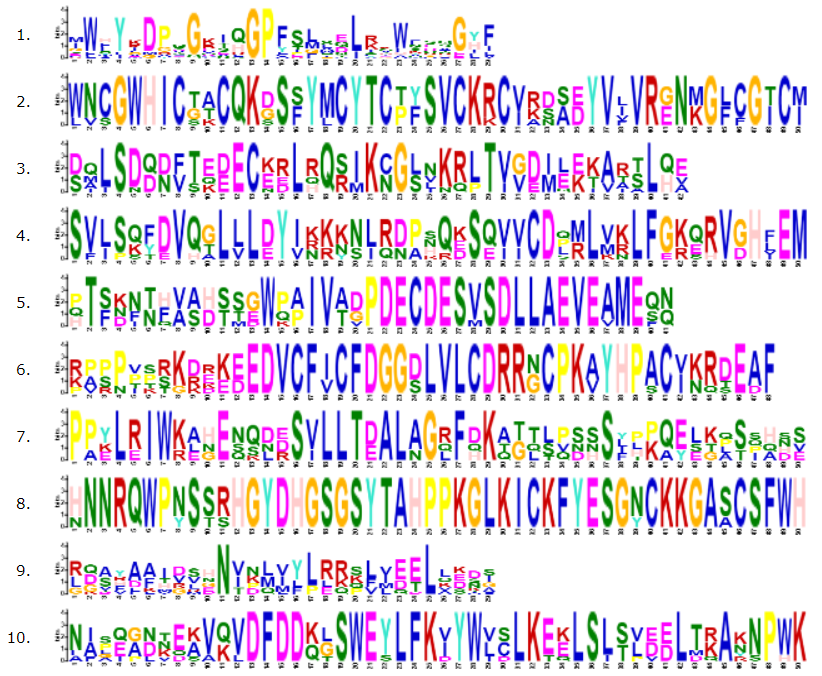

Supplement: Supplementary file 1 [file genes-14-00224-s001.zip › Supplementary figures/Figure S4.png]

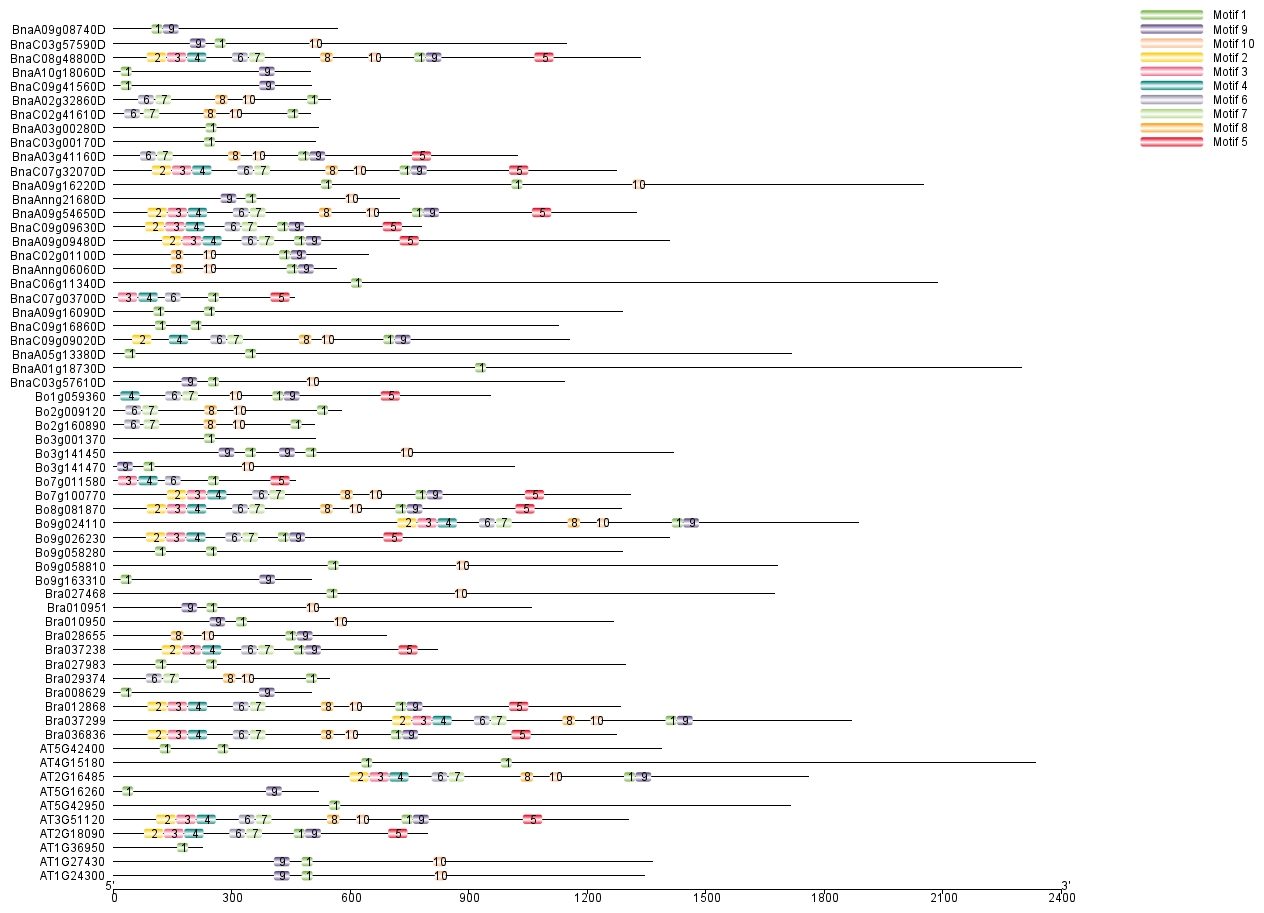

Supplement: Supplementary file 1 [file genes-14-00224-s001.zip › Supplementary figures/Figure S5.jpg]

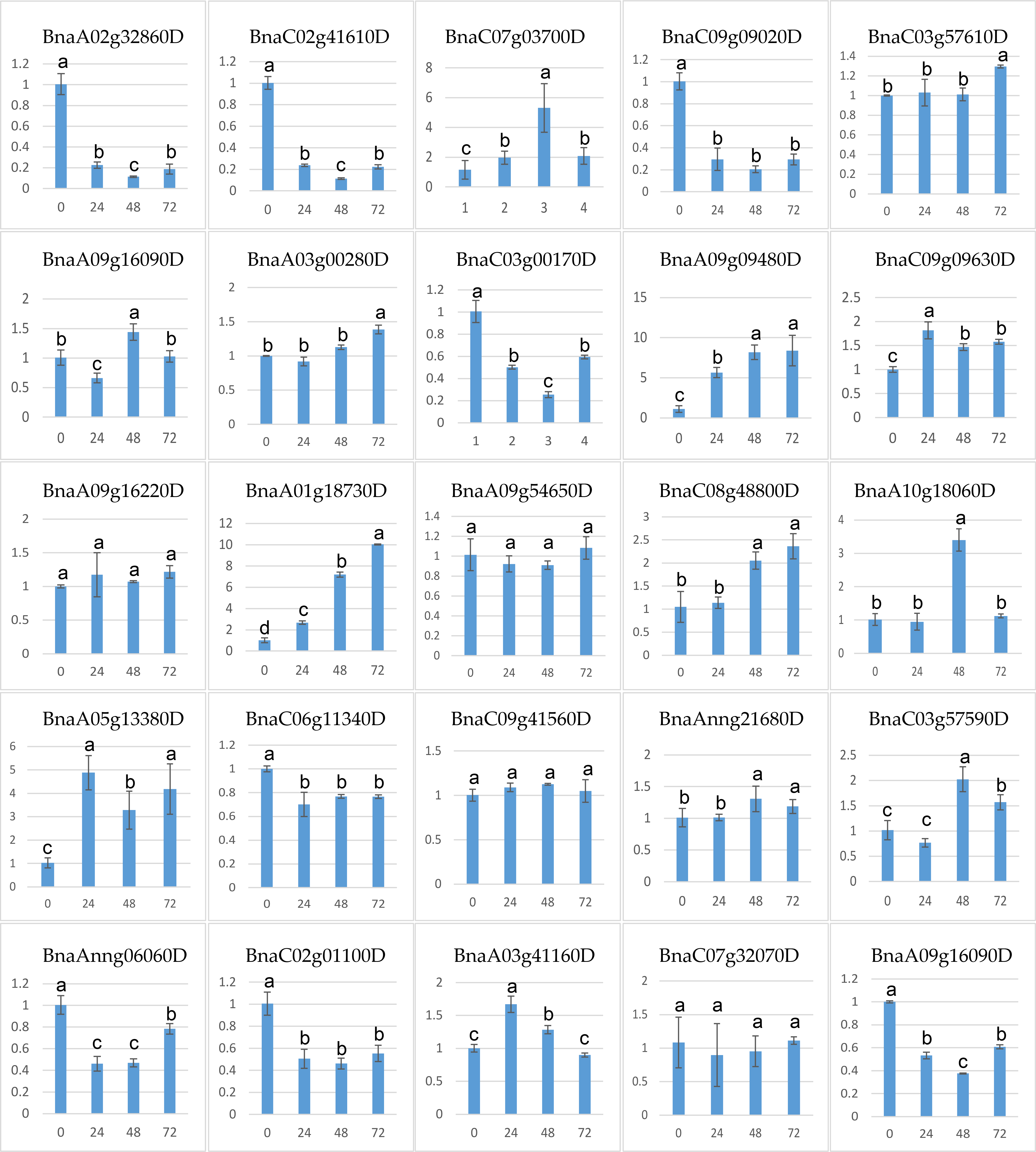

Supplement: Supplementary file 1 [file genes-14-00224-s001.zip › Supplementary figures/Figure S6.jpg]
